# Efficacy of disintegrating aspirin in two different models for acute mild-to-moderate pain: sore throat pain and dental pain

**DOI:** 10.1007/s10787-015-0253-0

**Published:** 2015-11-24

**Authors:** Voelker M, Schachtel BP, Cooper SA, Gatoulis SC

**Affiliations:** Bayer HealthCare, Leverkusen, Germany; Schachtel Research Company, Jupiter, FL USA; Clinical Trial Consultant, Palm Beach Gardens, FL USA; Bayer HealthCare, Whippany, NJ USA

**Keywords:** Aspirin, Acetylsalicylic acid, Paracetamol, Acetaminophen, Onset of action, Dental pain, Sore throat pain, Acute pain, Pain model, Stopwatch

## Abstract

A recently developed fast-release aspirin tablet formulation has been evaluated in two different pain models. The dental impaction pain model and the sore throat pain model are widely used for assessing analgesia, including acute mild-to-moderate pain. Both studies were double-blind, randomized, parallel group and compared a single dose of 1000 mg aspirin with 1000 mg paracetamol and with placebo and investigated the onset and overall time course of pain relief. Speed of onset was measured by the double-stopwatch method for time to meaningful pain relief and time to first perceptible pain relief. Pain intensity and pain relief were rated subjectively over a 6-h (dental pain) and 2-h (sore throat pain) time period. In both models fast-release aspirin and commercial paracetamol were statistically significantly different from placebo for onset of action, summed pain intensity differences and total pain relief. Meaningful pain relief was achieved within a median of 42.3 and 42.9 min for aspirin and paracetamol, respectively, in the dental pain model. The corresponding numbers in sore throat pain were 48.0 and 40.4 min. All treatments in both studies were safe and well tolerated. No serious adverse events were reported and no subject was discontinued due to an adverse event. Overall the two studies clearly demonstrated efficacy over placebo in the two pain models and a comparable efficacy and safety profile between aspirin and an equivalent dose of paracetamol under the conditions of acute dental pain and acute sore throat pain.

*Trial registration* These trials were registered with ClinicalTrials.gov, registration number: NCT01420094, registration date: July 27, 2011 and registration number: NCT01453400, registration date: October 13, 2011.

## Introduction

Aspirin (acetylsalicylic acid, ASA) is a widely used non-steroidal anti-inflammatory drug (NSAID) with analgesic, antipyretic and anti-inflammatory properties. It is commonly used in the nonprescription setting for the treatment of mild-to-moderate pain (Hersh et al. 2000) and fever (Bachert et al. 2005). Since its introduction several new formulations have been developed and marketed, e.g., granules, effervescent tablets, and chewable tablets. Recently, a fast release, solid tablet formulation has been developed and its efficacy for pain following third molar extraction determined. This new formulation contains sodium carbonate as a disintegrant and small particles of the active ingredient, both contributing to fast dissolution, absorption and consequent onset of action (Voelker and Hammer 2012; Cooper and Voelker 2012).

The fast-release tablet strength is 500 mg of aspirin. Two standard analgesic efficacy studies investigated the onset and overall time course of pain relief compared to an equivalent strength of a conventional non-rapidly disintegrating paracetamol formulation in two different pain models. In these studies two tablets of aspirin (1000 mg) were compared with a corresponding dose of paracetamol (2 tablets, 1000 mg). The pain models investigated were dental pain and sore throat pain due to upper respiratory tract infection (URTI). Both models are commonly used for assessing analgesia in acute mild-to-moderate pain (Food and Drug Administration 1988; European Medicine Agency 2002).

The dental impaction pain model has been widely used in the development and assessment of analgesics since the mid-1970s and has several inherently favorable attributes (Cooper and Beaver 1976). In this model, otherwise healthy patients undergo surgical removal of impacted (imbedded in the jaw) third molars. The surgery which involves creating a soft tissue flap, removal of some alveolar bone and sectioning of the impacted tooth or teeth is usually done under local anesthesia with or without short-acting conscious sedation. The subsequent onset of postsurgical pain is predictable and occurs within 1–3 h of the surgery as the effects of the local anesthetic wears off. Patients can be recruited to the study and screened in advance of the elective surgery thus allowing potential confounding treatments to be minimized. The dental impaction pain model is unique amongst the pain models used to study nonprescription analgesics as the time and intensity of pain onset is predictable and other confounding factors are well controlled. In addition, the intensity of the pain resulting from the dental surgical procedure can be prospectively estimated based on which teeth are surgically removed, the number of impacted teeth extracted and the extent (how badly imbedded) of the impactions (Cooper and Desjardins 2010).

The dose–response data generated from studies in the dental impaction pain model also closely parallel the same range of doses that have been evaluated in the sore throat pain model (Schachtel 1991). Like the dental pain model, the sore throat pain model is a tried-and-true analgesic assay (Hersh et al. 2000; European Medicine Agency 2002) used worldwide since the early 1980s (Schachtel et al. 1984) by different investigators (Blagden et al. 2002; Benrimoj et al. 2001). It is based on the most common type of human pain, sore throat, experienced since childhood throughout adulthood. As such, patients are familiar with the condition and find it easy to rate (in terms of pain intensity) and describe (in terms of sensory qualities, in particular, and throat function). As in the dental pain model, subjects in a sore throat pain study tend to be young adults in a university setting; they are excellent “test-takers”, adept at documenting responses, and generally otherwise healthy, thus avoiding interference by co-morbid medical conditions or medications that can confound pharmacologic evaluations in a controlled clinical trial. The procedural components of the sore throat pain model (also referred to as the pharyngitis pain model) have been well-described, implementing basic principles of study design, objective confirmation of the pain-producing condition (on the Tonsillo-Pharyngitis Assessment) (Schachtel et al. 2007); specification of homogeneous, well-characterized subjects before treatment, sensitive instruments to rate pain, its relief and functional outcomes (Schachtel et al. 2014a, b). The construct of a sore throat study has been applied to single-dose evaluations, multiple-dose evaluations over 24 h and 7 days (Blagden et al. 2002; Benrimoj et al. 2001; Schachtel et al. 1988, 2007, 2011, 2014a, b), onset-of-action determinations (Schachtel et al. 1994, 2014a, b), discrimination between doses of the same analgesic (Schachtel et al. 2002, 2010) and between different analgesics (Schachtel et al. 1984) and analgesic adjuvants (Schachtel et al. 1991), revealing the upside assay sensitivity of the model.

## Methods

Both of the reported studies were single-center, randomized, double-blind, double-dummy, placebo-controlled, parallel-group, and assessed the comparative onset of action of the fast-release aspirin tablet in subjects with either postoperative dental pain or sore throat pain. Eligible subjects in either study were randomized in 2:2:1 fashion to either a single dose of aspirin tablets (Aspirin^®^, Bayer HealthCare, Germany) equivalent to 1000 mg, paracetamol caplets (Tylenol^®^ Extra Strength, McNeil Consumer Healthcare, US) equivalent to 1000 mg, or placebo. The placebos used in the two studies were Aspirin^®^ matching placebo and Tylenol^®^ Extra Strength matching placebo.

The studies were conducted at investigative sites located in the United States. The studies were conducted in accordance with Good Clinical Practice guidance, and each protocol was approved by an institutional review board (IRB). All participants provided written informed consent.

### Dental pain

This study enrolled 510 healthy, ambulatory male and female volunteers, 16–45 years of age, scheduled to undergo surgical extraction of impacted third molars and who had moderate-to-severe postoperative pain.

After experiencing postsurgical pain of at least moderate severity, between approximately 1 and 4 h after surgery, subjects rated pain intensity (PI) on an 11-point Numerical Pain Intensity Rating Scale (subjects must have reported at least 5 on this scale), a 0–10 ordinal scale with endpoint of 0 = no pain and 10 = very painful, and on a 4-point Categorical Pain Intensity Scale (0 = no pain, 1 = mild pain, 2 = moderate pain, 3 = severe pain).

Eligible subjects were randomly assigned to one of the three treatment groups previously described and were administered their assigned dose of study drug. Subjects were included if they were scheduled to under surgical extraction of either two mandibular partial bony impactions or one mandibular full bony alone or in combination with a mandibular partial bony impaction, soft tissue impaction, or erupted third molar. Maxillary third molars could have been removed regardless of impaction level. Subjects were excluded if they had any clinically significant concomitant disease, including asthma, chronic sinusitis, or nasal abnormalities. In addition, subjects were excluded if they had a history of bleeding disorders, including gastrointestinal bleeding or perforation related to previous NSAID therapy. Subjects were also excluded if they had used any analgesics within 5 days of surgery, or any caffeine-containing substances within 12 h of study drug administration.

Pain intensity and pain relief (PR) were rated by subjects at 5, 10, 15, 20, 25, 30, 35, 40, 50, and 60 min and 1.5, 2, 3, 4, 5, and 6 h after dosing and immediately prior to the use of any rescue medication. The double-stopwatch method was used to calculate time to “first perceptible PR” and “first perceptible PR confirmed” (stopwatch 1) and time to “meaningful PR” (stopwatch 2). The study coordinator started the 2 stopwatches at the time of dosing.

The primary endpoint was defined as time to meaningful pain relief, while secondary endpoints included:

- time to first perceptible PR,
- time to first perceptible PR confirmed,
- PR and sum of Pain Intensity Difference (PID) scores at 5, 10, 15, 20, 25, 30, 35, 40, 50, and 60 min and at 1.5, 2, 3, 4, 5, and 6 h after dosing,
- Summed Pain Intensity Differences (SPID_0–2_) (summed, time-weighted PR from 0 to 2 h after dosing),
- total pain relief (TOTPAR_0–2_) (summed, time-weighted total PR from 0 to 2 h after dosing),
- summary scores for SPID_0–4_, TOTPAR_0–4_, SPID_0–6_, and TOTPAR_0–6_,
- time to first intake of rescue medication and the cumulative proportion of subjects taking rescue medication by time point,
- global assessment of PR at 6 h after dosing or immediately before the first intake of rescue medication.

Approximately 500 subjects (200 subjects per active treatment group and 100 subjects for the placebo treatment group) provided 90 % power to detect a treatment difference between aspirin and paracetamol for time to treatment onset at a 2-sided significance level of 0.05. This sample size was derived under the assumptions that the proportion of subjects who would experience meaningful relief by 6 h was 76 % for the aspirin group and 60 % for the paracetamol group.

### Sore throat pain

This study enrolled 177 otherwise healthy, ambulatory male and female volunteers, 18 years of age and older who presented to the clinic within 6 days of onset of sore throat pain due to upper respiratory tract infection (URTI). Eligible subjects must have had a baseline sore throat PI score of ≥60 mm on the 100 mm Sore Throat Pain Intensity Scale (STPIS), and had a score ≥5 on the Tonsillo-Pharyngitis Assessment (TPA).

The TPA is an index that provides descriptions and gradations of the clinical signs of tonsillo-pharyngitis and is based upon previous work by Schachtel et al. (2007). The TPA takes into account ratings (each on a 0–3 point scale) of the intensity of each of seven clinical features of tonsillo-pharyngitis: oral temperature, oropharyngeal color, size of tonsils, number of oropharyngeal exanthemas (vesicles, petechiae or exudates), cervical adenopathy (largest size of anterior cervical lymph nodes), cervical adenopathy (number of anterior cervical lymph nodes) and cervical adenitis (maximum tenderness of some anterior cervical lymph nodes). The sum of these seven ratings comprises the total score for the TPA (i.e., 0–21).

Subjects presented to the clinic within 6 days of onset of sore throat pain due to URTI and were screened for participation in the study. Eligible subjects were stratified by baseline PI (≤80 vs >80 mm) and randomly assigned in 2:2:1 fashion to one of the three previously described treatment groups and were administered their assigned dose of investigational product. In keeping with its onset-of-action objective, study assessments were conducted over 2 h, Subjects rated their PI at 15, 30, 45, 60, 75, 90, 105 and 120 min after dosing, and, if applicable, at the time meaningful PR had been achieved and immediately prior to the use of any rescue medication. The double-stopwatch method was used to record analgesic onset of action as described for the dental pain study.

Approximately 175 completing subjects (70 per active treatment group and 35 for the placebo treatment group) provide 80 % power to detect a treatment difference between aspirin and paracetamol for the time to meaningful pain relief at a two-sided significance level of 0.05. This sample size was derived under the assumption that aspirin would provide approximately a 40 % reduction in the time to meaningful pain relief as compared to paracetamol.

## Results

### Dental pain

The intent-to-treat (ITT) population of study 1 consisted of 510 patients. There were no group differences with respect to age, gender and baseline pain intensity (Table 1). The average age of the subjects was approximately 18 years. About 60 % of subjects on average had severe pain and about 40 % moderate pain at baseline. Mean pain intensity on the 11-point pain intensity scale was 7.9 at baseline. Two molars had been removed in more than 70 % of subjects and almost all subjects had a full bony impaction (>90 %).

**Table 1:** Summary of demographics and baseline characteristics

Study	Variable	Aspirin	Paracetamol	Placebo
Dental pain study (study 1)	ITT population	204	204	102
	Age, years [mean (SD)]	18.2 (1.87)	18.2 (2.04)	18.2 (2.03)
	Gender ratio, male:female (%)	43.1:56.9	51.5:48.5	48.0:52.0
	11-point intensity [mean (SD)]	7.9 (1.31)	7.9 (1.30)	7.9 (1.27)
	Categorical pain intensity (%)			
	Moderate	40.7	36.3	41.2
	Severe	59.3	63.7	58.8
	Number of molars removed (%)			
	1	4.9	3.9	6.9
	2	71.6	74.0	73.5
	3	5.4	5.4	6.9
	4	18.1	16.7	12.7
	Tooth sites (%)^a^			
	Left upper third molar	51.5	51.5	35.3
	Left lower third molar	51.0	51.5	34.3
	Right upper third molar	65.7	66.2	76.5
	Right lower third molar	68.6	66.7	79.4
	Impaction score (%)^a^			
	Erupted in tissue	2.5	2.5	0
	Broken soft tissue	6.4	3.9	1.0
	Partial bony impaction	36.3	41.7	40.2
	Full bony impaction	92.2	90.2	94.1
Sore throat pain study (study 2)	ITT population	71	70	36
	Age, years [mean (SD)]	19.5 (1.77)	19.4 (1.44)	19.7 (1.22)
	Gender ratio, male:female (%)	57.7:42.3	45.7:54.3	50.0:50.0
	Pain intensity score on 100 mm VAS [mean (SD)]	75.1 (8.28)	75.9 (8.37)	74.1 (9.83)
	Pain intensity (%)			
	Greater than 80 mm	26.8	25.7	27.8
	Less than or equal to 80 mm	73.2	74.3	72.2
	Days having sore throat [mean (SD)]	2.6 (1.31)	2.7 (1.14)	2.5 (1.03)
	Tonsillo-pharyngitis assessment [mean (SD)]	9.2 (2.95)	10.1 (2.94)	9.8 (2.97)

^a^Patients may had more than 1 affected tooth site or impaction score

**Fig. 1:**
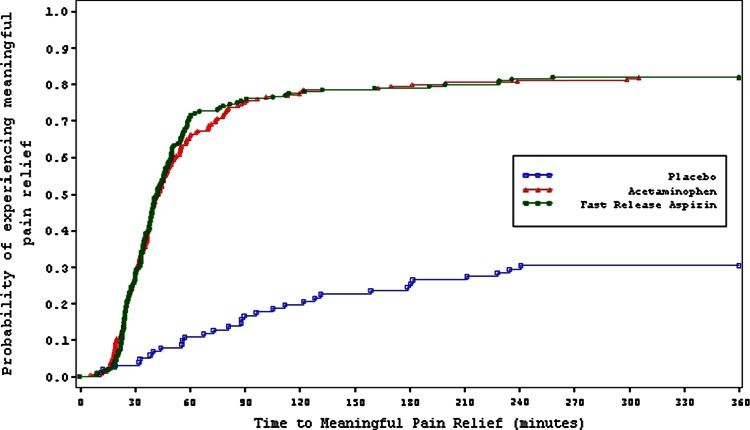
Dental pain study (study 1) Kaplan–Meier plot of time to meaningful pain relief. *Green curve* aspirin, *red curve* paracetamol, *blue curve* placebo

The Kaplan–Meier curve of the primary efficacy endpoint, time to meaningful pain relief, showed statistically both the aspirin group and the paracetamol group separated from the placebo group (*p* < 0.001) (Fig. 1; Table 2). Descriptively there was no difference between the two active groups (*p* = 0.945). A single dose of either 1000 mg aspirin or 1000 mg paracetamol achieved meaningful pain relief within a median of 42.3 and 42.9 min, respectively. For placebo, no median time to meaningful pain relief could be calculated due to the number of censored observations. Median time to first perceptible pain relief was obtained within 17.2 min for aspirin, 15.0 min for paracetamol and 27.3 min for placebo. Differences for active vs placebo were statistically significant (*p* < 0.001); whereas, differences between actives were not (*p* = 0.392).

**Table 2:** Time to relief and efficacy results

Study	Variable	Aspirin	Paracetamol	Placebo
Dental pain study (study 1)	Median time to FPR, min (95 % CI)	17.2 (15.0, 19.1)	15.0 (14.6, 16.1)	27.3 (20.0, 35.0)
	*p* value vs aspirin	–	0.392	<0.001
	*p* value vs paracetamol	–	–	<0.001
	% of subjects achieved FPR	96.6	97.1	70.6
	% of subjects censored	3.4	2.9	29.4
	Median time to MR, min (95 % CI)	42.3 (38.8, 46.5)	42.9 (38.8, 48.2)	NA (NA, NA)
	*p* value vs aspirin	–	0.945	<0.001
	*p* value vs paracetamol	–	–	<0.001
	% of subjects achieved MR	81.9	81.9	30.4
	% of subjects censored	18.1	18.1	69.6
	SPID 0–2 h, mean (SD)	2.4 (1.5)	2.6 (1.4)	0.3 (1.3)
	*p* value vs aspirin	–	0.267	<0.001
	*p* value vs paracetamol	–	–	<0.001
	SPID 0–4 h, mean (SD)	4.4 (3.3)	5.4 (3.2)	0.8 (3.3)
	*p* value vs aspirin	–	0.002	<0.001
	*p* value vs paracetamol	–	–	<0.001
	SPID 0–6 h, mean (SD)	5.9 (5.1)	7.9 (4.9)	1.3 (5.5)
	*p* value vs aspirin	–	<0.001	<0.001
	*p* value vs paracetamol	–	–	<0.001
	TOTPAR 0–2 h, mean (SD)	4.3 (1.9)	4.5 (1.8)	1.4 (1.6)
	*p* value vs aspirin	–	0.283	<0.001
	*p* value vs paracetamol	–	–	<0.001
	TOTPAR 0–4 h, mean (SD)	8.0 (4.4)	9.5 (4.2)	3.0 (4.1)
	*p* value vs aspirin	–	<0.001	<0.001
	*p* value vs paracetamol	–	–	<0.001
	TOTPAR 0-6 h, mean (SD)	11.0 (7.0)	14.0 (6.7)	4.5 (6.8)
	*p* value vs aspirin	–	<0.001	<0.001
	*p* value vs paracetamol	–	–	<0.001
Sore throat pain study (study 2)	Median time to FPR, min (95 % CI)	33.3 (28.9, 41.7)	30.5 (25.7, 35.1)	90.8 (45.8, NA)
	*p* value vs aspirin	–	0.523	<0.001
	*p* value vs paracetamol	–	–	<0.001
	% of subjects achieved PR	91.5	91.3	52.8
	% of subjects censored	8.5	8.7	47.2
	Median time to MR, min (95 % CI)	48.0 (39.7, 56.9)	40.4 (35.3, 53.7)	NA (NA, NA)
	*p* value vs aspirin	–	0.772	<0.001
	*p* value vs paracetamol	–	–	<0.001
	% of subjects achieved MR	73.2	79.7	30.6
	% of subjects censored	26.8	20.3	69.4
	SPID 0-1 h, mean (SD)	15.0 (12.6)	16.1 (14.6)	4.2 (8.6)
	*p* value vs aspirin	–	0.632	<0.001
	*p* value vs paracetamol	–	–	<0.001
	SPID 0-2 h, mean (SD)	48.0 (33.3)	47.1 (3.4)	13.4 (22.0)
	*p* value vs aspirin	–	0.869	<0.001
	*p* value vs paracetamol	–	–	<0.001

*FPR* first perceptible pain relief, *MR* meaningful relief, *CI* confidence interval, *SPID* summed pain intensity differences, *TOTPAR* total pain relief, *NA* not available

The SPID and TOTPAR results were consistent overall. SPID and TOTPAR at 2 h were comparable between aspirin and paracetamol and both were statistically superior to placebo (*p* < 0.001). At 4 and 6 h SPID and TOTPAR for both actives were statistically significantly different from placebo (all *p* < 0.001). There were statistically significant differences between aspirin and paracetamol showing a non-confirmatory trend favoring paracetamol (*p* = 0.002 and <0.001 for SPID 4 and 6 h; *p* < 0.001 for TOTPAR 4 and 6 h).

Both active treatments were safe and well tolerated. No serious adverse events occurred and no subject was discontinued due to an adverse event.

### Sore throat pain

One hundred and seventy-seven subjects were treated when conducting the sore throat study (study 2). Demographic and baseline characteristics were similar across the 3 treatment groups (Table 1). The overall mean age was 19.5 years. At baseline, the overall mean pain intensity score was 75.2 mm, and most subjects (about 73 %) had a baseline pain intensity score less than or equal to 80 (moderate pain). The baseline upper respiratory tract infection symptoms reported most frequently included sore throat (100.0 %), swollen neck glands (78.0 %), tender neck glands (76.8 %), drowsiness and lack of energy (53.1 % each), and headache (51.4 %), and were reported by a similar number of subjects across the three treatment groups. Subjects had a sore throat for an overall mean of 2.6 days at study entry and a mean tonsillo-pharyngitis score of 9.6.

**Fig. 2:**
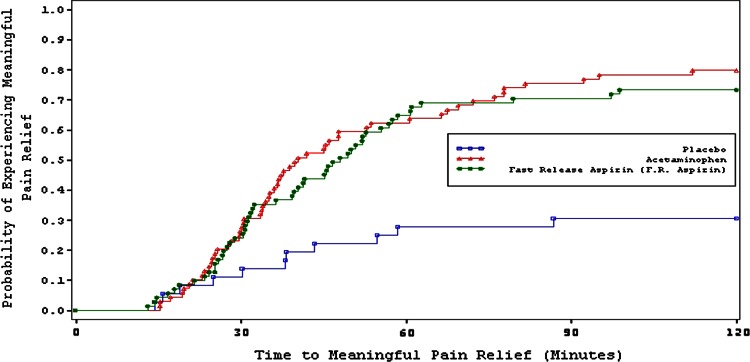
Sore throat pain study (study 2) Kaplan–Meier plot of time to meaningful pain relief. *Green curve* aspirin, *red curve* paracetamol, *blue curve* placebo

The primary efficacy endpoint was time to meaningful pain relief. The corresponding Kaplan–Meier figure is presented in Fig. 2. Mean time to meaningful pain relief was 48.0 min for aspirin, 40.4 min for paracetamol and was not achieved for placebo within the observation period of 2 h (Table 2). Differences to placebo were statistically significant (*p* < 0.001); whereas, the difference between aspirin and paracetamol was not (*p* = 0.772). For time to first perceptible pain relief, differences to placebo were significant (*p* < 0.001); whereas, differences between aspirin and paracetamol were not significant (*p* = 0.523) (aspirin 33.3 min, paracetamol 30.5 min, placebo 90.8 min) (Table 2).

Summed pain intensity differences measured as SPID0-1h and SPID0-2h showed consistently significant differences between aspirin and placebo (*p* < 0.001) and between paracetamol and placebo (*p* < 0.001). Differences between aspirin and paracetamol were not significant (*p* = 0.632 and 0.869, respectively) (Table 2).

The treatments were safe and well tolerated. No serious adverse events were reported and no subject was discontinued due to an adverse event.

## Safety and tolerability

Adverse events reported in the two studies are shown in Table 3. In the dental pain study, the percentages of subjects with at least one adverse event were 9.8, 10.8 and 17.6 % for aspirin, paracetamol and placebo, respectively. The corresponding numbers in the sore throat study were 18.3, 14.3 and 33.3 %, respectively. The numbers of adverse events reported for the gastrointestinal system organ class were 7.4 % (aspirin), 8.3 % (paracetamol) and 9.8 % (placebo) and 4.2 % (aspirin), 4.3 % (paracetamol) and 8.3 % (placebo).

**Table 3:** Treatment-emergent adverse events (AE) reported by at least 1 % of patients overall (safety population)

Study	Variable	Aspirin	Paracetamol	Placebo
Dental pain study (study 1)	Number of patients	204	204	102
	Total number of AEs	30	36	30
	Number of subjects with at least 1 AE [*n* (%)]	20 (9.8)	22 (10.8)	18 (17.6)
	Gastrointestinal disorders	15 (7.4)	17 (8.3)	10 (9.8)
	Nausea	11 (5.4)	16 (7.8)	10 (9.8)
	Vomiting	8 (3.9)	9 (4.4)	4 (3.9)
	Nervous system disorder	4 (2.0)	4 (2.0)	7 (6.9)
	Headache	4 (2.0)	2 (1.0)	3 (2.9)
	Dizziness	0	1 (0.5)	4 (3.9)
Sore throat pain study (study 2)	Number of patients	71	70	36
	Total number of AEs	20	11	13
	Number of subjects with at least 1 AE [*n* (%)]	13 (18.3)	10 (14.3)	12 (33.3)
	Gastrointestinal disorders	3 (4.2)	3 (4.3)	3 (8.3)
	Nausea	1 (1.4)	1 (1.4)	1 (2.8)
	Vomiting	2 (2.8)	0	0
	Enlarged uvula	1 (1.4)	0	1 (2.8)
	Respiratory, thoracic, and mediastinal disorders			
	Oropharyngeal pain	7 (9.9)	2 (2.9)	5 (13.9)
	Cough	3 (4.2)	1 (1.4)	2 (5.6)
	Nasal congestion	1 (1.4)	0	2 (5.6)
	Wheezing	1 (1.4)	1 (1.4)	1 (2.8)
		3 (4.2)	0	0
	Infections and infestations	4 (5.6)	1 (1.4)	4 (11.1)
	Laryngitis	1 (1.4)	0	2 (5.6)
	Upper respiratory tract infection	3 (4.2)	0	0
	Tonsillitis	0	1 (1.4)	1 (2.8)
	General disorders and administration site conditions	2 (2.8)	0	1 (2.8)
	Pain	2 (2.8)	0	0
	Nervous system disorders	1 (1.4)	2 (2.9)	0
	Headache	1 (1.4)	1 (1.4)	0

## Discussion

For the treatment of acute mild-to-moderate pain, patients seek fast onset of relief (Hersh et al. 2000; Schachtel et al. 1999). Recently a fast release aspirin formulation, characterized by the inclusion of sodium carbonate as a disintegrant and smaller active ingredient particles, has been developed and approved in many countries for the treatment of mild-to-moderate acute pain. This formulation has improved dissolution and pharmacokinetics and consequently faster onset of action compared to the original aspirin formulation (Voelker and Hammer 2012; Cooper and Voelker 2012). The time to meaningful pain relief is an important clinical endpoint providing a very relevant advantage for patients with mild-to-moderate pain (Schachtel et al. 1999). In the recent dental pain study (Cooper and Voelker 2012), a time of 49.4 min for the fast-release formulation compared to 99.2 min for the original formulation has been determined, indicating a twofold improvement in analgesic onset. However, it is important to provide evidence that this endpoint can be achieved in different pain models. In addition to acute dental pain, the sore throat pain model is also well-regarded by researchers and regulators as an appropriate general pain model (Hersh et al. 2000; European Medicine Agency 2002). Furthermore, relative performance of analgesic active ingredients and analgesic medicinal products is of high interest with respect to decision making in analgesia.

The two present studies confirm the results seen in the earlier study (Cooper and Voelker 2012). Time to meaningful pain relief for fast-release aspirin was determined to be 42.3 min in dental pain. Small differences to the results seen in the earlier study (Cooper and Voelker 2012) may be explained by study and population variability. Also in the study with patients having sore throat pain and treated with the fast-disintegrating aspirin, the time to meaningful pain relief (48 min) is consistent with the dental pain study. Generally, the results of the present dental pain and sore throat pain studies confirm the data of the earlier dental pain study (Cooper and Voelker 2012). Neither the dental pain study nor the sore throat pain study showed a statistically significant difference between aspirin 1000 mg and paracetamol 1000 mg in any determination of onset.

Conventional aspirin is absorbed in the stomach and in the upper small intestine, but the upper small intestine is considered to be the main absorption site because of the poor solubility of aspirin in the acidic pH of the stomach and the small absorption surface of the stomach mucosa, as opposed to the much greater surface of the small intestine (Schroer 2009). The new fast-disintegrating formulation is characterized by its small particle size and the sodium carbonate disintegrant. This theoretically supports absorption in the stomach by enhancing disintegration of the tablet as well as increasing surface area due to the smaller particle size. In addition, the new aspirin formulation may accelerate gastric emptying. This factor may be clinically important since sympathetic stimulation due to pain or its associated trauma may slow gastric emptying (Jamali and Kunz-Dober 1999; Jamali and Aghazadeh-Habashi 2008). Interestingly, the time to maximum plasma concentration (*T*_max_) for the new aspirin formulation is comparable to soluble highly buffered aspirin formulations (Voelker and Hammer 2012; Kanani et al. 2015) which also may enhance gastric emptying. However, this is very speculative and needs to be more intensively investigated.

## Conclusions

The two efficacy studies described in this paper further support the efficacy and safety of the novel fast-release aspirin formulation. This formulation is characterized by the inclusion of sodium carbonate as a disintegrant and smaller active ingredient particles. Efficacy in two different acute pain models has been shown. Patients with dental pain and patients with sore throat pain showed significantly faster and better pain relief when treated with aspirin compared to patients treated with placebo. Generally, the two studies demonstrated an equivalent efficacy and safety profile for equivalent doses of aspirin and paracetamol (1000 mg) under the conditions of acute dental pain and acute sore throat pain.
